# Cancer Molecular Evolution

**DOI:** 10.1007/s00239-015-9695-7

**Published:** 2015-08-20

**Authors:** David Posada

**Affiliations:** Department of Biochemistry, Genetics and Immunology and Institute of Biomedical Research of Vigo (IBIV), University of Vigo, 36310 Vigo, Spain

By far, the majority of studies in molecular evolution have focused on genetic change across one or more generations. Much less is known about the genetic changes that occur during the life time of an individual, or somatic evolution, of which cancer is probably the best-known example. Cancer is an adaptive evolutionary process in which distinct genetic clones compete for space and resources (Cairns 1975; Greaves and Maley 2012; Nowell 1976).

Modern cancer biology and genomics have validated the evolutionary nature of cancer, which has attracted much attention in recent years (Burrell and Swanton 2014; Gerlinger et al. 2014). Not surprisingly, cancer genomics has unveiled a significant amount of intratumor heterogeneity in most tumor types (Burrell et al. 2013; Michor and Polyak 2010; Swanton 2012). However, but logically, most studies in cancer genomics have been mostly concerned with the identification of the genetic and epigenetic changes that lead to cell transformation, tumor growth, metastasis and drug resistance, and much less with molecular evolutionary aspects. Despite a relatively rich literature on cancer evolutionary dynamics (Michor et al. 2004; Sottoriva et al. 2011), little is known about the evolutionary mechanisms that drive tumor progression at the molecular and cellular level, and evolutionary insights in cancer are based, for the most part, on mathematical models of carcinogenesis (Beerenwinkel et al. 2015).

Fortunately, the chance to obtain a more quantitative understanding of cancer molecular evolution is here. Decreasing NGS costs and the already large amount of genomic data made available by the International Cancer Genome Consortium (https://dcc.icgc.org) or The Cancer Genome Atlas (http://cancergenome.nih.gov) should facilitate detailed studies on the molecular evolution of cancer. Still, the paramount opportunity for molecular evolutionary studies in cancer will be the one provided by single-cell genomics techniques (Wang and Navin 2015), which open wide the door for the application of the conceptual and analytical machinery of molecular evolution. In particular, population genomics and phylogenomics, which are mostly based on individualized data (e.g., DNA or protein sequences), will prove to be critical tools in this regard.

There are multiple fundamental questions in cancer that might be at least partially answered under a sound molecular evolution framework. For example, what are the relative roles of selection and genetic drift? While the presumption is that selection is the solely driving force in cancer, this might depend on the particular scenario (Sottoriva et al. 2015). In fact, estimates derived from mathematical model fitting suggest that the selective advantage of driver mutations can be as low as 0.4–1 % (Beerenwinkel et al. 2007; Bozic et al. 2010). Basic parameters—such as tumor effective population size, cell generation times or cell turnover—for understanding the role of genetic drift in cancer progression have not yet been formally measured (Merlo et al. 2006). Indeed, the characterization of the adaptation process and the role of neutrality have always been fundamental themes in molecular evolution (Lachance and Tishkoff 2013; Savolainen et al. 2013), and existing methods should be used to interrogate cancer genomic data.

In addition, statistical models of evolution will be useful in testing hypotheses about potentially different substitution processes acting in a tumor, or to measure tumoral evolutionary rates from genomic data. Relaxed molecular clocks could be utilized to estimate whether a metastasis was established early or late after transformation, or the temporal dynamics of tumor growth (see for example Zhao et al. 2014). Also, tumor genealogies could help in understanding tumor demographics in tempo and space, for example as typically done in viral epidemiology. Likewise, models of tumor growth including the cancer stem cell model (see Navin and Hicks 2010) could be formally compared under a phylogenetic framework using available statistical tests. Existing methods for the identification of convergent evolution might help to pinpoint new “driver genes”, particularly for non-coding regions. Moreover, ancestral character reconstruction techniques could be exploited to infer the genomic composition of the initial cell that originated a primary tumor or a metastasis, and the order of the different genomic events that took place. Major evolutionary aspects like the role and extent of tumor population structure, admixture, or different forms of gene flow—not just metastases but also the movement of cells within tumors, or into contiguous regions—currently not well understood, could also benefit from the use of standard tools in population genomics.

Indeed, somatic cancer evolution differs from germline evolution in fundamental ways (Sidow and Spies 2015). Most importantly, somatic evolution is asexual, proceeding through cell mitosis and therefore without chromosomal segregation or crossing over. The fact that the whole cancer genome is non-recombining implies that genetic hitchhiking and clonal interference should be common (see for example Neher 2013), but also that the variance of the different estimates from a single locus will be huge, so some care is need in this regard. Furthermore, all significant variations in cancer occur *de novo* in each individual—with the exception of a few transmissible cancers—meaning that the time scale and the level of divergence will be very narrow. Importantly, many parameter estimates or tests in molecular evolution assume neutrality, so the robustness of these methods to the presence of selection should be assessed before their application in cancer.

Understanding cancer evolution can have significant applications, such as more robust predictive biomarkers to improve personalized treatment and prevent drug resistance (Jamal-Hanjani et al. 2015; McGranahan and Swanton 2015). Evolutionary signatures such as clonal expansions or mutational diversity could help predicting cancer progression (Maley et al. 2006; Mengelbier et al. 2015; see also Neher et al. 2014). Evolutionary biology concepts and tools remain underused in cancer biology (Aktipis et al. 2011; Shibata 2012). The availability of genomic data, in particular that from single cells, should help change this.
